# Microbial communities in freshwater used for hydraulic fracturing are unable to withstand the high temperatures and pressures characteristic of fractured shales

**DOI:** 10.1099/acmi.0.000515.v3

**Published:** 2023-04-21

**Authors:** Sophie L. Nixon, Alvaro M. Plominsky, Natali Hernandez-Becerra, Christopher Boothman, Douglas H. Bartlett

**Affiliations:** ^1^​ Manchester Institute of Biotechnology, University of Manchester, Manchester, UK; ^2^​ Department of Earth and Environmental Sciences, University of Manchester, Manchester, UK; ^3^​ Marine Biology Research Division, Scripps Institution of Oceanography, University of California San Diego, San Diego, CA, USA

**Keywords:** hydraulic fracturing, high pressure, fractured shale microbial communities, fracturing fluid additives

## Abstract

Natural gas is recovered from shale formations by hydraulic fracturing, a process known to create microbial ecosystems in the deep subsurface. Microbial communities that emerge in fractured shales include organisms known to degrade fracturing fluid additives and contribute to corrosion of well infrastructure. In order to limit these negative microbial processes, it is essential to constrain the source of the responsible micro-organisms. Previous studies have identified a number of potential sources, including fracturing fluids and drilling muds, yet these sources remain largely untested. Here, we apply high-pressure experimental approaches to assess whether the microbial community in synthetic fracturing fluid made from freshwater reservoir water can withstand the temperature and pressure conditions of hydraulic fracturing and the fractured shale environment. Using cell enumerations, DNA extraction and culturing, we show that the community can withstand high pressure or high temperature alone, but the combination of both is fatal. These results suggest that initial freshwater-based fracturing fluids are an unlikely source of micro-organisms in fractured shales. These findings indicate that potentially problematic lineages, such as sulfidogenic strains of *Halanaerobium* that have been found to dominate fractured shale microbial communities, likely derive from other input sources into the downwell environment, such as drilling muds.

## Data Summary

The authors confirm all supporting data, code and protocols have been provided within the article or through supplementary data files, available in the online version of this article. All sequencing data generated in this work are available at the National Center for Biotechnology Information (NCBI) Sequencing Read Archive under BioProject PRJNA889574.

## Introduction

Hydraulic fracturing involves the high-pressure injection of freshwater fluids into shale formations deep in the terrestrial subsurface to stimulate the production of natural gas. Fracturing fluids typically contain sand and additives, included to hold open new fractures and enhance the efficiency of the process, respectively [1]. The process of hydraulic fracturing is now understood to create new microbial ecosystems kilometres beneath the Earth’s surface [2, 3]. The microbial communities that inhabit fractured shale formations are typically low in diversity and capable of withstanding the high salinities that develop in the months following hydraulic fracturing [3].

Some microbial processes in fractured shale ecosystems can impact negatively on shale gas extraction. These include the degradation of fracture fluid additives and the production of corrosive sulfide and organic acids as metabolic by-products [4–6]. In addition, biofilm formation may lead to fracture clogging, leading to lower gas yields [2, 3, 7, 8]. Some predominant fractured shale taxa are capable of multiple negative processes. For instance, strains of *Halanaerobium* that dominate late-stage fractured shale communities recovered from shale gas wells in the USA can couple the fermentation of organic polymers to the reduction of thiosulfate, yielding corrosive organic acids and sulfide [4, 9]. Biocides, such as glutaraldehyde and quaternary ammonium compounds, are frequently added to fracturing fluids to prevent microbial activity from impacting on shale gas recovery [1]. However, numerous studies have demonstrated these to be of limited effectiveness against fractured shale communities [2, 10–14]. The common practice of reusing produced fluids in subsequent hydraulic fracturing operations may even select for biocide resistance in persistent taxa [13]. It is therefore critical to constrain the origins of the micro-organisms in fractured shale communities in order to effectively control these negative processes through more targeted approaches.

Microbial communities that develop in fractured shales are remarkably similar in composition, despite their recovery from geographically and geologically distinct formations. Almost all fractured shale communities studied to date exhibit an enrichment of halotolerant strains driven by increasing salinity, leading to a low-diversity, predominantly anaerobic community that is often dominated by *Halanaerobium* [2, 4, 10, 14–19]. Further, a number of shale formations such as the Barnett formation are considered ‘paleopasteurized’, where diagenesis has subjected the formation to temperatures above the upper temperature limit for microbial life of 122 °C [14]. Evidence of downwell microbial activity in these shale formations therefore suggests that the micro-organisms observed in fractured shale communities are introduced during shale gas extraction activities, rather than being native to the shale.

There are a number of likely sources of micro-organisms in shale gas extraction, including drilling fluids, equipment, additives, hydraulic fracturing fluid and the subsurface itself [2, 20]. Most studies to date have focused on the community composition of pre- and post-fracturing fluids [2, 10, 14, 15, 21]. It is evident from these studies that the communities recovered in flowback and production fluids differ markedly from those that are injected. This shift has been observed in fluids from numerous fractured shale formations, suggesting that these communities develop *in situ* in response to the prevailing physical and geochemical conditions. Although prior studies have hypothesized that the source of these micro-organisms is the freshwater-based input fluid [2, 17], little work has been done to assess this, and hence the sources of fractured shale taxa remain unclear.

For micro-organisms in input fluids to survive the hydraulic fracturing process, they must withstand the very high pressures of fluid injection and the subsequent moderately high-temperature, high-pressure conditions of the fractured shale environment. We hypothesized that these conditions represent a bottleneck in microbial survival of freshwater communities introduced during hydraulic fracturing. Here, we use high-pressure experiments to test whether a microbial community in simulated freshwater-based injection fluids can withstand the high-pressure and moderately high-temperature conditions of the hydraulic fracturing process and of the fractured shale environment that results. We show that freshwater micro-organisms can withstand either high pressure or moderately high temperature, but not both, indicating their inability to withstand these conditions during hydraulic fracturing. We discuss the implications of these findings in the management of unwanted microbial activity during shale gas extraction.

## Methods

### Sample collection

Water samples were collected from Dovestone Reservoir in the north of England on 28 June 2018 (53.530°N, 1.975°W). This reservoir was chosen as a representative source of freshwater used in proposed hydraulic fracturing activities in the Bowland Shale in the north of England. Samples were collected from the edge of the reservoir, accessed near the footpath. This part of the reservoir was <1 m in depth, and samples were taken from the near surface of the water. A total of 10 l of reservoir water was collected in autoclaved (121 °C for 21 min) 1 l wide-mouth leakproof HDPE bottles (Fisher Scientific, Loughborough, UK). Collection bottles were filled to the brim, the lids wrapped twice with parafilm and stored in a 10 °C cold room within 4 h of collection prior to being shipped to Scripps Institute of Oceanography within 48 h of collection. Upon arrival, water samples were stored at 4 °C until experiments were initiated, 7 days after collection.

### Experimental setup

To simulate freshwater-based hydraulic fracturing fluids, reservoir water was used as the base of a synthetic fracturing fluid (SFF). This water also served as the sole source of micro-organisms in the experiments. SFF was designed to mimic the organic composition of typical hydraulic fracturing fluids [22, 23]. Hydraulic fracturing fluids also commonly contain inorganic additives; however, these are typically used as cross-linkers and breakers for the viscosity modifier used, or biocides [1]. Since the viscosity of the SFF was not modified for these purposes, and the response of the native microbial communities was the main objective of the experiments, inorganic additives were left out. The composition of SFF used in these experiments (l^−1^ of reservoir water) was 0.56 g guar gum, 0.43 g citric acid, 0.34 g choline chloride, 0.8 ml ethylene glycol, 0.12 ml hydrotreated petroleum distillate and 2.2 ml methanol (90 % w/v). The pH was adjusted to 7.1–7.4 using 5M KOH.

Organic additives were added to SFF the same day that experiments were initiated. To prevent the contamination of the experiments with micro-organisms from the laboratory environment, all additives were UV-screened in a microbial cabinet for 30 min prior to their addition to reservoir water (NuAire AireGard ES NU-201 Series Workstation). The UV lamp in this cabinet emits light within the 253.7 nm range, and at the time of the experiment was generating 40–49 µW cm^−1^. In real hydraulic fracturing operations, additives would not be sterile, and may in fact represent an important source of micro-organisms that are introduced to fractured shales. However, the chemicals used in these experiments were laboratory grade, and had been handled in a high-pressure microbiology laboratory, and therefore could otherwise contribute pressure-adapted micro-organisms to the experiments that would be unrepresentative of hydraulic fracturing operations.

Experiments were designed to assess the effects of high pressure and high temperature (together and independently) on the native freshwater microbial community. Pressure–temperature regimes were chosen to mimic those of a hydraulically fractured shale (35 MPa, 50 °C). The additional effect of the higher pressures of the hydraulic fracturing process itself were tested by the additional 15 min of 70 MPa, prior to post-fracturing conditions for the remainder of incubations (referred to as 70+35 MPa, 50 °C). The effects of high temperature (50 °C) and high pressure (35 MPa) alone were also tested, along with a control experiment at room temperature and pressure. In total, five experimental setups were prepared, including controls, as summarized in Table 1. All treatments were tested in triplicate, where each replicate was incubated in a single pressure vessel.

**Table 1.** Details of formation conditions simulated in high-pressure experiments. Experiments were designed to mimic the pressure (*P*) of hydraulic fracturing, and the pressure and temperature (*T*) conditions of hydraulically fractured shales, as well as controls to test the effects of each in isolation.

Setup	Conditions simulated	Pressure/temp.
1	Hydraulic fracturing pressure+fractured shale *T* and *P*	70 MPa (15 min) and 35 MPa/50 °C
2	Fractured shale *T* and *P*	35 MPa/50 °C
3	Fractured shale *P* only	35 MPa/22 °C
4	Fractured shale *T* only	0.1 MPa/50 °C
5	Ambient *T* and *P* control	0.1 MPa/22 °C

Prior to incubation in pressure vessels, SFF was transferred to 250 ml capacity Teflon bags (SealPAK, Kapak, Minneapolis, MN, USA) and heat-sealed closed without headspace. These custom-made bags were presterilized by filling with 100 % ethanol for 5 min before decanting and being left to dry in a microbial cabinet for 1 h. Heat-sealed SFF-filled 250 ml bags were transferred to pressure vessels [24] and the void space was filled with deionized water prior to pressurization. Each vessel contained one SFF bag, and each experimental setup was conducted in triplicate. Experimental setups conducted at ambient pressure were prepared in bags as described above, but stored within the HDPE bottles used for water collection and incubated at room temperature or 50 °C, as required. One litre of reservoir water was left unamended with SFF additives and stored at 4 °C for the duration of the incubations to serve as a baseline for cell numbers and community composition (‘no chemical 4 °C control’). All experiments were incubated for 2 weeks in the dark.

### Follow-on culturing

To assess whether members of the community had survived being subject to high pressure, 10 % v/v of experimental end points from the three high-pressure (35 MPa at 50 °C, 35 MPa at room temperature and 70+35 MPa at 50 °C) were inoculated into fresh SFF in nitrogen-flushed sterile serum vials and incubated at 30 °C for 115 days. Growth was determined visually by increased turbidity (preliminary experiments of fresh reservoir water in SFF had led to the development of visible turbidity in 48 h) and production of organic acid metabolic by-products monitored as described below.

### Analytical methods

Cell numbers were quantified at the end of incubations using flow cytometry. Hoechst 33 342 dye (10 μg ml^−1^, Invitrogen Molecular Probes, Eugene, OR, USA) was added to 500 μl samples to a final concentration of 1 μg μl^−1^ and left to stain in the dark for 1 h. Flow cytometry was conducted on 10 μl of stained samples using the ZE5 Cell Analyzer (BioRad, Hercules, CA, USA) equipped with the small-particle detection module. Hoechst 33 342 was excited off the 355 nm laser (50 mW) and fluorescence was collected through a 447/6 nm band-pass filter. Sample delivery was by a calibrated peristaltic pump, allowing for precise measurement of absolute counts. Owing to time constraints, it was not possible to quantify cell numbers in the ‘no chemical 4 °C control’ at the start (*T*=0) of incubations. The baseline cell number in incubations was therefore difficult to assess. However, triplicate samples from day 3 of this control, in addition to experiments conducted at ambient pressure (50 °C at atmospheric pressure; room temperature at atmospheric pressure), were fixed with 37 % w/v formaldehyde to a final concentration of 3 % and cell numbers were quantified by flow cytometry as above.

Production of putative metabolic by-products (volatile fatty acids; VFAs) was monitored in follow-on cultures using ion chromatography, as described previously [25]. Briefly, samples (1 ml) were extracted using a sterile syringe and needle, and centrifuged (13 000 ***g*** for 7 min, room temperature). The supernatant was diluted 100-fold with deionized water. Diluted samples were injected into a Dionex ICS5000 Dual Channel Chromatograph via a Dionex AS-AP autosampler (Thermo Fisher Scientific, Waltham, MA, USA). Molecule separation was achieved by passing samples through a 250×0.4 mm Dionex AS11-HC capillary column (4 um pore size) at a flow rate of 0.015 ml min^−1^ at 3400 psi pressure. The mobile phase was KOH, electronically injected to produce a gradient from 1 to 40 mM.

Mean end-point cell numbers and VFA concentrations between experiments were assessed for statistical significance using a Student *t*-test (two-tailed, type 2, critical value of 0.05), and corresponding *P* values discussed below.

### DNA extraction

To assess the microbial community composition of reservoir water used in high-pressure experiments, a 450 ml sample of the *T*=3 ‘no chemical 4 °C control’ was filtered through 0.2 and 0.1 µm filters and DNA was extracted using the DNeasy PowerWater kit (MO BIO Laboratories, Inc., Carlsbad, CA, USA) according to the manufacturer’s instructions. This is referred to as 'inoculum' in the results presented below. Samples of experimental end points from all experimental setups were processed for microbial community analysis immediately after incubations ended. Owing to the presence of guar gum in SFF, samples could not be filtered to concentrate biomass. Instead, 300 ml (pooled from the three replicates) from each experiment was concentrated into a pellet by centrifugation at 7000 ***g*** for 30 min, 15 °C. After initial centrifugation, pellets and 5 ml supernatant from each replicate were combined and centrifuged again as before. Pooled concentrated samples were frozen at −80 °C prior to DNA extractions. DNA was extracted from thawed pooled sample pellets as follows: lysis of biomass for 55 °C for 30 min using a mixture of 4M guanidine thiocyanate, 2 % sarkosyl, 50 mM EDTA, 40 µg ml^−1^ proteinase K and 15 % β-mercaptoethanol. This was followed by homogenization using a Mini-Beadbeater-8 (BioSpec Products, USA) for 20 s with 0.1 mm silica beads, followed by extraction with one volume of phenol : chloroform : isoamyl alcohol (25 : 24 : 1). Double-stranded (ds) DNA was quantified using the Qubit dsDNA High Sensitivity assay according to manufacturer’s instructions (Invitrogen), and the concentration of dsDNA was read using a Qubit Fluorometer (Thermo Fisher Scientific) with a detection range of 0.2–100 ng. The concentration of DNA recovered from each sample is herein referred to as the ‘DNA yield’.

### 16S rRNA gene sequencing

16S rRNA genes were amplified via PCR by targeting the V4 hypervariable region (forward primer, 515F, 5′-GTGYCAGCMGCCGCGGTAA-3′; reverse primer, 806R, 5′-GGACTACHVGGGTWTCTAAT-3′) [26] and sequenced using the Illumina MiSeq platform (2×250 bp paired-end sequencing; Illumina, San Diego, CA, USA) [27, 28]. The Roche FastStart High Fidelity PCR System (Roche Diagnostics Ltd, Burgess Hill, UK) was used to perform 50 µl PCR reactions as follows: initial denaturation at 95 °C for 2 min; 36 cycles of 95 °C for 30 s; 55 °C for 30 s; 72 °C for 1 min and a final extension step at 72 °C for 5 min. PCR products were subsequently purified and normalized to approximately 20 ng each with the SequalPrep Normalization kit (Fisher Scientific, Loughborough, UK). PCR amplicons from all samples were pooled in equimolar ratios, and the sequencing run performed using 4pM sample library, spiked with 4pM PhiX to a final concentration of 10 % (following [29]).

Demultiplexed paired-end sequences were processed using QIIME2 version 2021.4 [30]. Denoising and amplicon sequence variants (ASVs) were obtained with the *DADA2* plugin [31]. Taxonomical assignment was obtained with the *q2-feature classifier* plugin [32] using the classify-sklearn naïve Bayes taxonomy classifier [33] against the Silva v138 99 % reference sequence database [30, 34]. ASVs classified as mitochondria or chloroplasts were flagged as potential artefacts and removed. Contaminant sequences identified in extraction and PCR controls were removed manually.

## Results

### Synthetic fracturing fluid high-pressure experiments

High-pressure experiments were designed to test the widely held assumption that hydraulic fracturing fluid source waters represent an important source of persistent micro-organisms that inhabit fractured shale environments in the hundreds of days after hydraulic fracturing has taken place. Experiments were conducted on a SFF at high pressure (70 or 35 MPa) and high temperature (50 °C) for a period of 2 weeks to assess the survival of a freshwater microbial community under the pressure and temperature regime of simulated hydraulic fracturing and the resulting hydraulically fractured shale conditions.

Upon arrival at Scripps Institution of Oceanography (prior to experiments being initiated), samples of the reservoir water were fixed for cell enumeration and found to contain 8.6×10^6^ (sd±6.3×10^6^) cells ml^−1^. It was not possible to enumerate cell numbers on the day experiments were initiated (*T*=0), but in the 7 days of storage at 4 °C between arrival and *T*=3 of the experiments, cell numbers in this water decreased to 3.2×10^5^ (sd±2.5×10^4^) cells ml^−1^ (no chemical 4 °C control), indicating a reduction in freshwater microbial community size during storage. We acknowledge that the time these samples spent in transit and storage is a limitation to our study since it is not possible to know which lineages may have been lost during this time. Cell numbers at *T*=3 of SFF high temperature (ambient pressure) experiments were higher at 4.9×10^5^ (sd±1.7^5^) cells ml^−1^, and higher still in SFF room temperature (ambient pressure) experiments at 2.7×10^6^ (sd±1.1×10^6^) cells ml^−1^. The higher cell numbers in SFF experiments compared with the ‘no chemical 4 °C control’ indicates that the microbial community was growing in these experimental setups, presumable using the SFF additives as substrates.

**Fig. 1.**
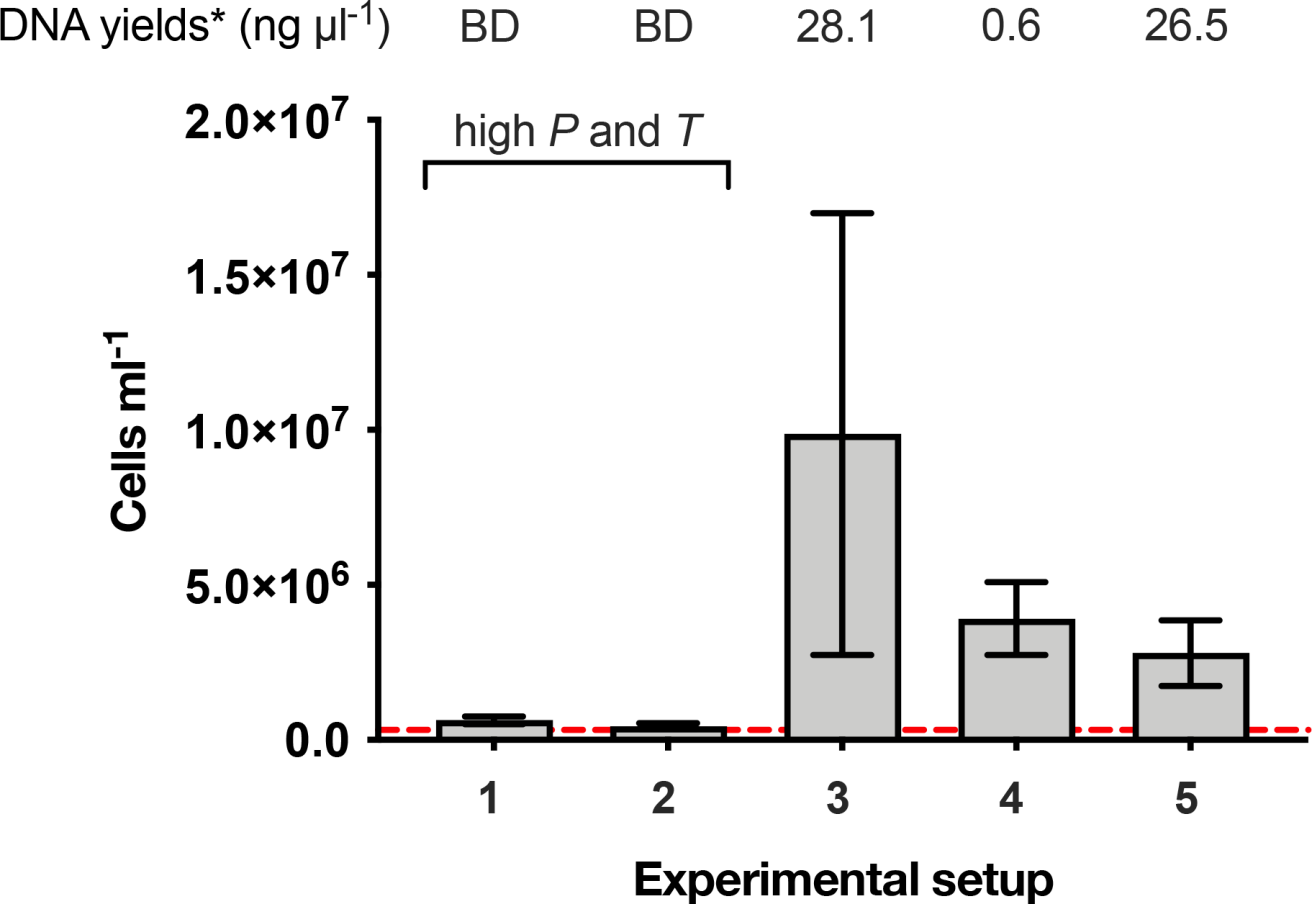
Cell numbers observed in high-pressure experimental end points. Details of experimental setups are given in Table 1. 1, 70 MPa (15 min)+35 MPa at 50 °C; 2, 35 MPa at 50 °C; 3, 35 MPa at 22 °C; 4, 0.1 MPa at 50 °C; 5, 0.1 MPa at 22 °C. The red dashed line indicates the cell numbers observed in the ‘no chemicals 4 °C control’, used here as a conservative baseline for starting numbers of cells in each experimental setup. These end points represent 14 days after experiments were started. Error bars represent the standard deviation of the mean of triplicate measurements. *, concentration of DNA recovered from 300 ml sample of experimental end points, where BD indicates that yields were below the limit of detection (0.5 ng ml^−1^).

Cell numbers and DNA yields of all experimental end points are summarized in Fig. 1. Average cell numbers were an order of magnitude lower at the end of combined high-temperature and high-pressure experiments compared with those incubated under high pressure only, high temperature only or room temperature and pressure conditions. End-point cell numbers in combined high-pressure, high-temperature experiments were similar to the *T*=3 ‘no chemicals 4 °C control’ baseline. Though not a true representation of cell numbers at the start of experiments, this baseline is considered a conservative estimate owing to the effect of refrigeration, with no added substrates already observed on the community, and the true population size is thought to be higher. In contrast, cell numbers in experiments with only high pressure (room temperature of ~22 °C) or high temperature (sea-level atmospheric pressure of 0.1 MPa) were significantly higher than this baseline (*P* value <0.01). Cell numbers in these experiments were also higher than in the room temperature and sea-level atmospheric pressure experiment. The results indicate that the combined effect of high pressure and high temperature inhibits population growth, yet either high pressure or high temperature alone stimulates population growth relative to the room temperature and pressure control.

Cell numbers alone cannot convey microbial community survival in response to incubation conditions. However, no detectable DNA was recovered from end points of combined high-pressure, high-temperature experiments, suggesting low microbial survival. Ample DNA was recovered from the inoculum and all other experimental end points from equal volumes of sample, thus it seems unlikely that the extraction method itself was responsible for the lack of quantifiable DNA.

**Fig. 2.**
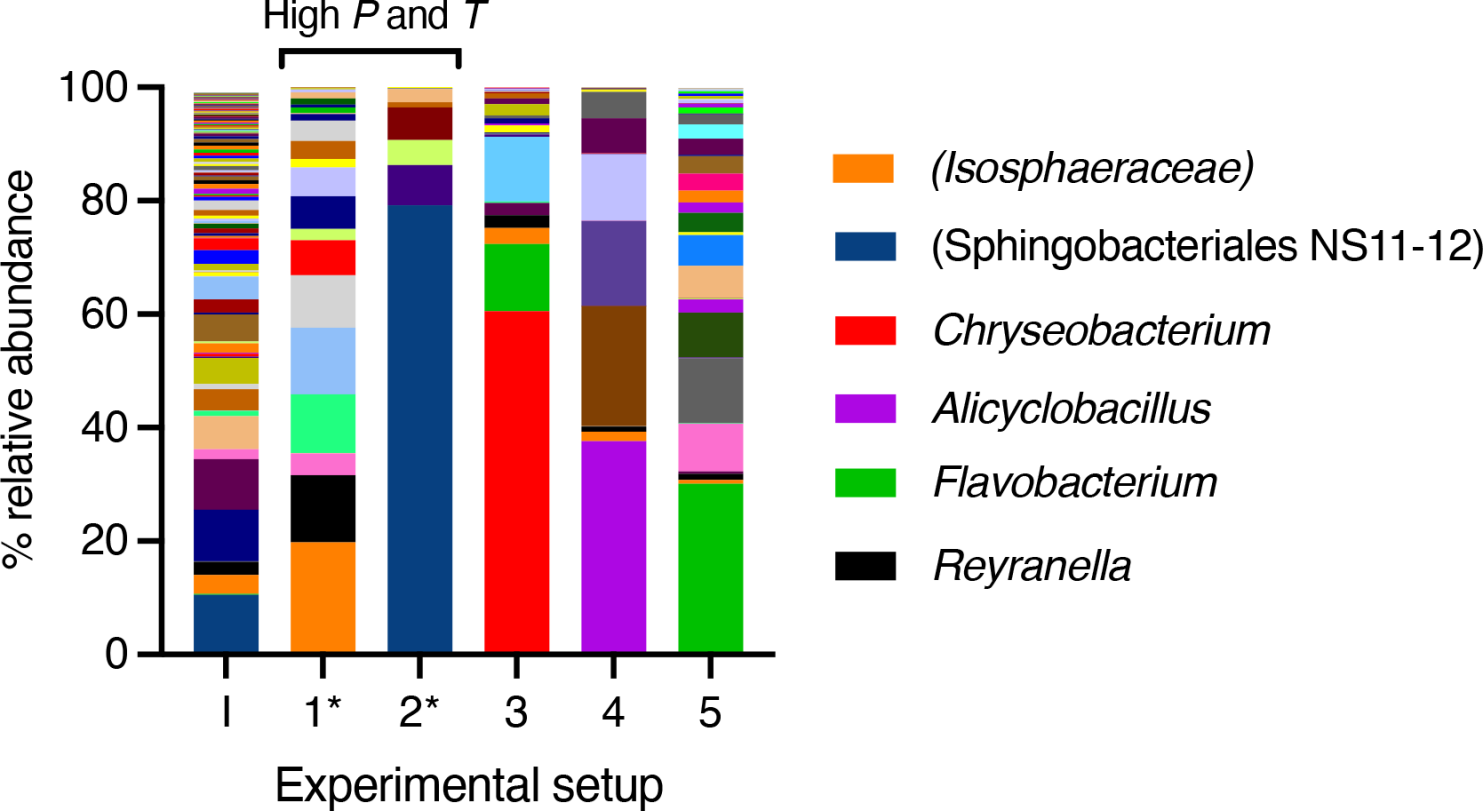
Microbial community composition in inoculum and experimental end points. Details of experimental setups are given in Table 1. I, inoculum; 1, 70 (15 min)+35 MPa at 50 °C; 2, 35 MPa at 50 °C; 3, 35 MPa at 22 °C; 4, 0.1 MPa at 50 °C; 5, 0.1 MPa at 22 °C. *, DNA yields recovered from 300 ml of experimental end points were below detection. Only prominent taxa across are shown in the legend. Lineages representing <0.1 % across all samples were omitted. ASVs identified in extraction and PCR controls were omitted prior to preparation of this figure.

Community composition in experimental end points compared to the baseline inoculum (*I*) is shown in Fig. 2 (see File S1, available in the online version of this article, for the full genus-level ASV table used to make this figure). Any ASVs detected in the sequencing data from extraction and PCR controls were removed manually from the dataset prior to further analysis and figure preparation. Despite the below-detection yields of DNA from the combined high-temperature and high-pressure experiment, diversity decreased as a result of all experimental treatments. End-point cultures from experiments subject to only high-temperature or high-pressure (but not both) were characterized by different community compositions. Taken together with cell number data (Fig. 1), this suggests that different populations were using SFF additives as substrates in each experimental setup, in response to the pressure–temperature regime they were subject to. For instance, most amplicon sequence variants (ASVs) in the 30 MPa room temperature experimental setup were assigned to the genus *Chryseobacterium *, whereas the dominant ASV lineage in the end-point 50 °C room pressure experiment was assigned to *Alicyclobacillus *. Interestingly, ASVs assigned to either genus were not detected in the inoculum. The community detected at the end of the room temperature and pressure incubations was more diverse than the other experimental conditions, and most of these ASVs were assigned to the genus *Flavobacterium* (Fig. 2).

**Fig. 3.**
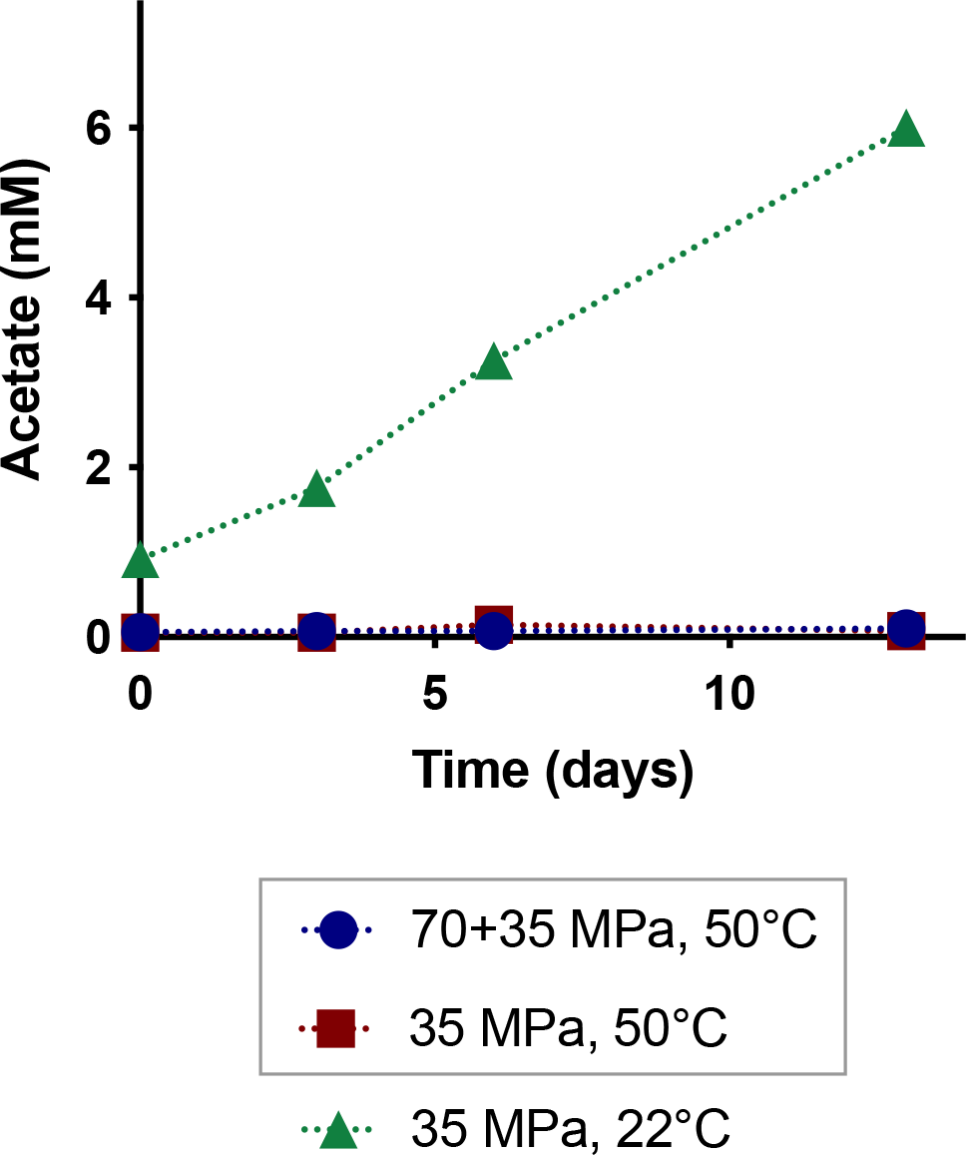
Acetate concentrations in follow-on culturing of high-pressure experimental end points. The grey box indicates the experimental incubations that were subject to combined high-pressure and high-temperature conditions. All follow-on culturing was conducted anaerobically at ambient conditions.

Follow-on culturing of subsamples taken from the end points of the three high-pressure experiments into fresh SFF was carried out to further investigate survivability. Preliminary tests with earlier batches of reservoir water in SFF gave rise to turbidity within 48 h compared to a blank control, indicating that the native community can utilize SFF additives for growth. However, no turbidity was observed with follow-on SFF cultures inoculated with combined high-pressure, high-temperature end points over a 115-day period, and no production of VFAs was observed. In contrast, turbidity and acetate production of up to 6 mM were observed in follow-on cultures inoculated with pooled high-pressure-only end point samples over the same period (Fig. 3). Together, these results indicate a lack of microbial survival after 2 weeks of incubation under combined high-pressure and high-temperature conditions.

## Discussion

Hydraulic fracturing of shale formations to recover onshore natural gas has been shown to create new microbial ecosystems in the deep terrestrial subsurface [2, 21]. Some commonly observed fractured shale taxa have the potential to cause problems, such as reservoir souring and microbiologically induced corrosion. In order to control these deleterious microbial processes, it is essential to constrain the sources of micro-organisms responsible in the shale gas extraction process. Previous studies have suggested that freshwater-based input fluids are a major source, while others have identified drilling muds as a possible origin [20, 35]. However, their microbial contributions to fractured shales remain poorly constrained.

Using a combination of high-pressure and high-temperature simulation experiments, we assessed the potential for freshwater-based injection fluids to inoculate fractured shale formations with micro-organisms. Synthetic fracturing fluid (SFF) made with freshwater reservoir water was subject to combined high-pressure and high-temperature conditions analogous to the hydraulic fracturing process and subsequently the fractured shale environment (Table 1). Three lines of evidence suggest that the conditions a freshwater community is subjected to during and after hydraulic fracturing are fatal.

Firstly, while cell numbers increased in high-pressure-only, high-temperature-only or room condition incubations, suggestive of growth on SFF additives, no increase in cell numbers was observed in combined high-pressure and high-temperature experimental setups (Fig. 1).

Secondly, no detectable yields of DNA were recovered from end points of high-pressure, high-temperature incubations, despite the recovery of DNA from equal volumes of other experimental end points and the baseline control (Fig. 2).

Thirdly, no growth was observed in follow-on cultures, in which fresh SFF was inoculated with high-pressure, high-temperature end points; in contrast, growth was observed in SFF inoculated with the high-pressure, room temperature end point. Taken together, these results suggest that micro-organisms native to freshwater used for high-pressure injection cannot survive the conditions of hydraulic fracturing or of the hydraulically fractured shale environment.

Previous research suggests that key fractured shale taxa are introduced in injected fluids. Daly *et al.* [2] recovered an identical *Halanerobium* genome from input and production fluids collected from a Marcellus shale gas well. This strain of *Halanerobium* became the dominant member of the fractured shale community after 82 days, and its presence in input fluid suggests that this organism was introduced in injection fluids. Based on 16S rRNA gene sequencing, other key taxa were detected in these input fluids, including *Marinobacter* and *Halomonadaceae *. However, one fifth of this input fluid was recycled produced water [2]. In light of findings from our hydraulic fracturing simulation experiments (Fig. 1), it is plausible that the taxa identified by Daly *et al.* [2] were introduced through the recycling of produced waters rather than the freshwater that dominates initial injection fluids. It is not clear what the ultimate origin of this lineage is in that system, but could feasibly include the drilling fluids and the subsurface itself.

It is perhaps not surprising that freshwater micro-organisms are not able to withstand the extreme conditions of hydraulic fracturing. Freshwater input fluids are typically inhabited by a diverse community of predominantly aerobic micro-organisms, not adapted to the anoxic high-pressure environments of the deep terrestrial subsurface. In contrast, it seems plausible that micro-organisms from the subsurface entrained in fluids during drilling can tolerate these conditions and inhabit deeper subsurface habitats. However, it is important to note that the investigations reported here do not include other components of the process that may contribute micro-organisms to fractured shales. Our high-pressure experiments were conducted on SFF made with sterilized chemicals. As such any contribution, however likely, of micro-organisms from fracturing fluid additives has not been accounted for. Similarly, because we have not assessed real input fluids used in hydraulic fracturing, we cannot account for the contribution of the infrastructure itself (including holding tanks, mixers and gas–water separators) to the microbial consortia previously observed in shale gas wells. The sources of fractured shale micro-organisms are likely numerous, and could include both chemicals and infrastructure. In addition, the now common practice of reusing production waters in subsequent fracturing fluids serves to recirculate already enriched taxa. This makes source tracking key taxa challenging.

To the best of our knowledge, the stimulation of our incubated communities in response to high pressure or high temperature alone has not previously been reported. High hydrostatic pressure has long been investigated for its potential use as a nonthermal food preservation method (see [36]) yet the pressures used are much higher than those applied here (typically 100 MPa or more), and it is widely reported to be an effective means of reducing (not stimulating) cell numbers [36]. Consistent with our findings, however, is the observation that combining high hydrostatic pressures (100 to 400 MPa) with high temperature (50 °C) is more effective at inactivating microbial cells than the application of high pressure alone, and this has been reported for both isolates [37] and microbial communities [38]. In a deep sea enrichment experiment, Marietou *et al*. applied similar pressures to those used here to crude oil-associated microbial communities using the same incubation approach, but at temperatures analogous to the deep sea environment (4 °C). In contrast to our findings, they observed a decrease in cell numbers with increasing pressure at this temperature [39].

Our community composition profiles suggest an enrichment of ASVs most closely associated with the *Chryseobacterium* in response to high pressure alone (relative to the 0.1 MPa treatment; Fig. 2). We were interested in whether this lineage had been observed in other high-pressure incubations, or is known to be piezotolerant. However, prior research on this lineage has focused on its role and multi-drug resistance in host microbiomes, human or otherwise [40], and we were unable to find reports of its presence in high-pressure environments or incubations. The end-point community incubated at high temperature (0.1 MPa) appeared to be enriched in members of the genus *Alicyclobacillus* (Fig. 2). Although the DNA yields from these incubations were lower than those for the control (Fig. 1), we note that strains of this spore-forming genus have long been known as spoilage bacteria that evade conventional heat treatment preservation methods in the beverage industry [41]. Further, *Alicyclobacillus montanus* was isolated from acidic hot springs in Colombia with an optimal temperature of ~45 °C [42], in line with the temperatures used in our incubations. Taken together, our findings suggest that lineages conventionally associated with pathogenesis (* Chryseobacterium *) and beverage spoilage (* Alicyclobacillus *) are likely to be widespread in the environment, including the freshwater used in these experiments. We infer that members of these lineages were able to proliferate under the high-pressure or high-temperature conditions of our incubations in response to their natural adaptations, highlighting their versatility when removed from their source habitat.

In summary, we have shown that freshwater micro-organisms from a freshwater reservoir, typical of a water source used in hydraulic fracturing, are not capable of withstanding the very high pressures of injection, or the combined high-temperature, high-pressure conditions of the fractured shale environment. As such, initial injection fluids do not appear to represent a significant source of micro-organisms that inhabit the fractured shale environment. These results suggest that other major inputs other than fracturing fluids are the source of dominant fractured shale taxa, for example the drilling muds used to drill the vertical and horizontal well bores, the chemical components of these fluids, the infrastructure used to access the shale formation, and the subsurface itself. Further study is needed to evaluate the relative survivability of micro-organisms in these components and their contribution to the fractured shale microbiome.

## Supplementary Data

Supplementary material 1
